# Acute podocyte injury is not a stimulus for podocytes to migrate along the glomerular basement membrane in zebrafish larvae

**DOI:** 10.1038/srep43655

**Published:** 2017-03-02

**Authors:** Florian Siegerist, Antje Blumenthal, Weibin Zhou, Karlhans Endlich, Nicole Endlich

**Affiliations:** 1 Department of Anatomy and Cell Biology, University Medicine Greifswald, Greifswald, Germany; 2 Department of Pediatrics and Communicable Diseases, University of Michigan, Ann Arbor, Michigan, USA

## Abstract

Podocytes have a unique 3D structure of major and interdigitating foot processes which is the prerequisite for renal blood filtration. Loss of podocytes leads to chronic kidney disease ending in end stage renal disease. Until now, the question if podocytes can be replaced by immigration of cells along the glomerular basement membrane (GBM) is under debate. We recently showed that in contrast to former theories, podocytes are stationary in the zebrafish pronephros and neither migrate nor change their branching pattern of major processes over 23 hours. However, it was still unclear whether podocytes are able to migrate during acute injury. To investigate this, we applied the nitroreductase/metronidazole zebrafish model of podocyte injury to *in vivo* two-photon microscopy. The application of metronidazole led to retractions of major processes associated with a reduced expression of podocyte-specific proteins and a formation of subpodocyte pseudocyst. Electron microscopy showed that broad areas of the capillaries became denuded. By 4D *in vivo* observation of single podocytes, we could show that the remaining podocytes did not *walk* along GBM during 24 h. This *in vivo* study reveals that podocytes are very stationary cells making regenerative processes by *podocyte walking* along the GBM very unlikely.

Podocytes, the visceral epithelial cells of the glomerulus, exhibit a unique 3D morphology with major processes and interdigitating foot processes connected by the slit diaphragm, the prerequisite for proper blood filtration of the kidney. Alterations of the podocyte morphology, like foot process effacement or loss of podocytes leads to chronic kidney disease (CKD), which often results in end stage renal disease (ESRD).

Since podocytes are postmitotic cells and unable to regenerate, there is an ongoing debate whether a kind of progenitor cell type – like e.g. the parietal epithelial cell of the Bowman’s capsule – is able to differentiate into podocytes. However, this hypothesis would assume that podocytes could migrate along the GBM to fill the gaps of the detached podocytes. To find out whether podocytes are per se able to migrate over longer distances, we studied the behavior of healthy podocytes *in vivo* in a living organism, the zebrafish larva. Recently, our group has shown by *in vivo* two-photon microscopy (2-PM) that podocytes of healthy zebrafish larvae are stationary and do not change the branching pattern of their major processes during 23 hours^1^. This observation was also found by Brähler *et al*. in mice. They recently demonstrated by 2-PM that transgenically labelled podocytes are stationary and do not migrate on the glomerular basement membrane (GBM)^2^.

Now, the question arose whether podocytes or their potential precursor cells start to migrate along the GBM if the neighboring podocytes detach and the GBM becomes naked. If progenitor cells would immigrate to the GBM, a coordinated lateral migration of podocytes toward the naked GBM would be necessary. To study this hypothesis, we studied the dynamics of single podocytes *in situ* and *in vivo* in the pronephros of living zebrafish larvae.

During the last decade, zebrafish became a very popular model organism for kidney research^3^,^4^ due to the similarity of the glomerulus of zebrafish, mouse and human. It has been shown in the past that podocytes of zebrafish larva develop the same foot process morphology and that the slit membrane is composed of podocyte-specific proteins like nephrin and podocin^5^. Due to the availability of transparent strains, the zebrafish larva is a powerful model for *in vivo* imaging, especially when being combined with *in vivo* 2-PM which enables *in vivo* microscopy to visualize structures that are located deeply in the organism. To find out whether podocytes start to migrate along the GBM if the neighbouring podocytes are detached and the GBM becomes naked we used a modified nitroreductase (NTR)/metronidazole (MTZ) model of cell ablation. In this injury model, the *E.coli* derived enzyme NTR is expressed under the podocyte-specific *nphs2* promotor together with a fluorescence protein in a transparent zebrafish strain^6^,^7^. When being applied to the tank water, the prodrug MTZ, which is converted by the NTR to a cytotoxin, leads to dose dependent apoptosis of NTR-expressing podocytes.

Summarizing, the highly selective podocyte injury model in zebrafish larvae provides convincing evidence that podocytes do not migrate along the naked GBM, which would be the prerequisite for a replacement of lost podocytes by immigrating progenitor cells.

## Results

### Establishment of a zebrafish disease model for *in vivo* observation by 2-PM

**Figure 1:**
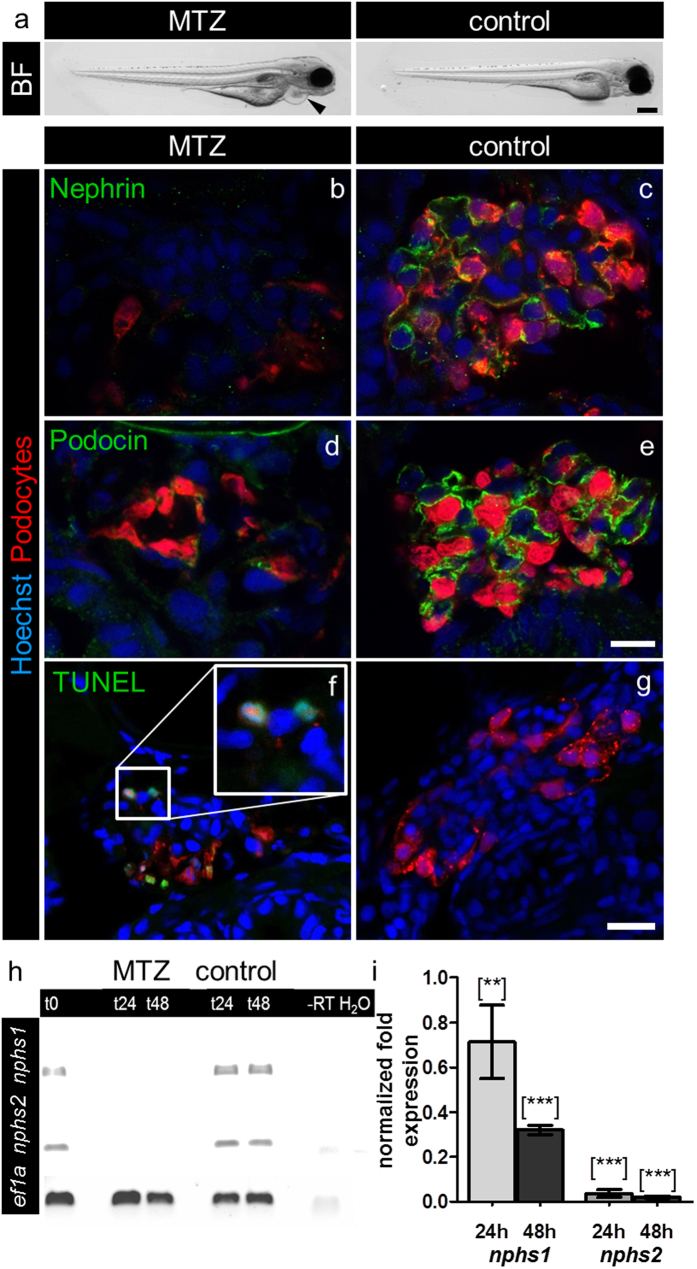
MTZ-treatment induces pericardial edema and downregulation of nephrin and podocin and podocyte apoptosis. Picture a shows (**a**) bright field (BF) image of a *Cherry* larva at 4 dpf after 24 hours 5 mM MTZ treatment that developed pericardial edema (arrowhead) in contrast to a 0.1% DMSO treated control larva exhibiting a normal phenotype. Panels b–e show confocal micrographs of immunofluorescence staining for nephrin and podocin of *Cherry* 4 dpf treated for 20 hours with 5 mM MTZ or 0.1% DMSO (control). Control glomeruli show pronounced staining for nephrin and podocin as well as a normal glomerular appearance, whereas glomeruli after MTZ treatment show decreased mCherry (podocytes) fluorescence as well as weak staining for nephrin and podocin (representative images of n = 3 individual experiments, scale bar represents 10 μm). Using TUNEL assay, we investigated the appearance of cell-death in MTZ treated (**f**) and control larvae (**g**). MTZ treated larvae show positive TUNEL staining, which co-localize with the nuclei of remaining podocytes (**f**, insert). The control larvae do not show glomerular TUNEL staining. The findings of the immunofluorescence staining could be verified on mRNA level by RT-PCR for *nphs1* and *nphs2* with e*ef1a1l1 (ef1a)* as reference gene. After 24 hours treatment in 10 mM MTZ (t24) and following 24 hours washout in E3 medium (t48) the band intensities after RT-PCR of *nphs1* and *nphs2* were significant weaker compared to control (panel h). Quantitative RT-PCR for both target genes with e*ef1a1l1* and *zgc:158463* as reference genes showed 0.71 (SD = 0.23) fold expression of *nphs1* and 0.04 fold expression (SD = 0.016) of *nphs2* compared to control after 24 hours 10 mM MTZ. After 24 hours regeneration in E3 medium the effect further increased as *nphs1* was expressed 0.32 fold (SD = 0.03) and *nphs2* 0.02 fold (SD = 0.004) compared to control (panel i). Mean value of n = 3 experiments, the error bars indicate standard deviation, [**] p < 0.01; [***] p < 0.001.

To study podocytopathy *in vivo* we used the podocyte-specific NTR zebrafish strain (Tg(*nphs2*:Eco.nfsB-mCherry)) which was crossed with the transparent zebrafish strain *Casper (mitfa*^*w2/w2*^*; roy*^*a9/a9*^) for *in vivo* imaging by 2-PM. The resulting strain (Tg(*nphs2*:Eco.nfsB-mCherry); *mitfa*^*w2/w2*^*; roy*^*a9/a9*^) was named *Cherry* (Supplementary Figure 1). After the exposure of *Cherry* larvae (at 3 days post fertilization (dpf)) to MTZ (5 mM) for 24 hours, they developed periocular and pericardial edema, a hallmark for proteinuria in zebrafish larvae (Fig. 1a, arrowhead).

Further, to identify the MTZ-induced changes of the glomerular filtration barrier we stained cross sections of *Cherry* larvae (20 μm) that were treated with MTZ for 20 hours with antibodies against nephrin and podocin. While MTZ treated larvae showed decreased amounts of nephrin and podocin (Fig. 1b,d), the control treated larvae (vehicle with 0.1% DMSO) showed no changes of the localization and expression of both proteins (Fig. 1c,e), which was verified by RT-PCR for *nephrin (nphs1*) and *podocin (nphs2*) mRNA with e*ef1a1l1* as reference gene. Whereas *nphs2* is exclusively expressed in podocytes, *nphs1* is also expressed in the brain. To minimize the *nphs1* mRNA signal from the brain we used decapitated zebrafish larvae. The expression of *nphs1* and *nphs2* mRNA was significantly decreased in MTZ treated Cherry larvae (Fig. 1h, t24) and did not increase after the recovery of the larvae in E3 medium for 24 hours (Fig. 1h, t48). These findings are consistent with the results obtained by qRT-PCR (e*ef1a1l1* and *zgc:158463* as reference genes) which showed a 0.71 fold (SD = 0.23) expression of *nphs1* and a 0.04 fold (SD = 0.016) expression of *nphs2* in MTZ treated larvae compared to control (Fig. 1i, 24 hours), respectively. Beside this, we found a 0.32 fold (SD = 0.03) expression of *nphs1* and 0.02 fold (SD = 0.004) expression of *nphs2* after recovery of the larvae in E3 medium (without MTZ) during 24 hours (Fig. 1i, 48 hours; n = 3 in three individual experiments), suggesting an irreversible loss of podocytes. To find out whether podocytes become apoptotic, we performed TdT-mediated dUTP-biotin nick end labeling (TUNEL) assay after 20 hours MTZ or control treatment. Figure 1f shows that several mCherry positive podocytes stained TUNEL positive in their nuclei (Fig. 1f, insert), whereas control treated larvae did not show glomerular TUNEL staining (Fig. 1g).

### MTZ treatment induces podocyte loss and denudation of the GBM

**Figure 2:**
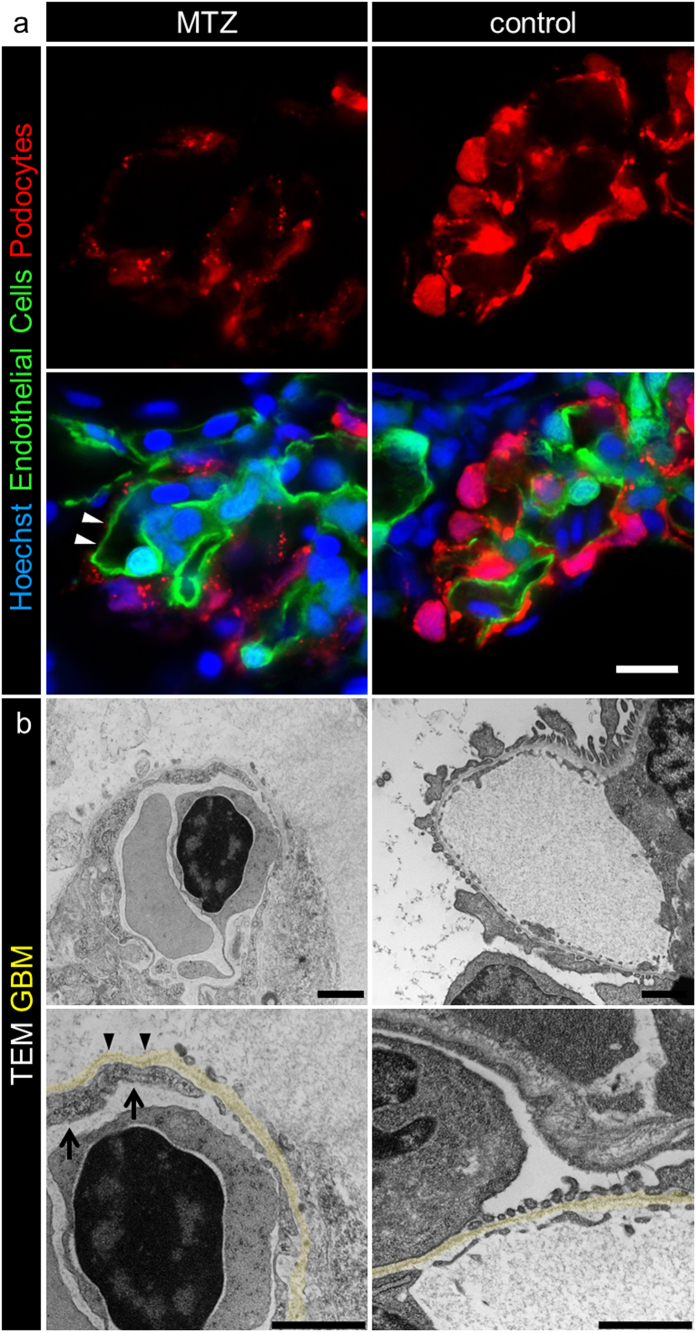
MTZ treatment leads to denudation of the GBM. Panel a shows denudation of glomerular capillaries after 16 hours exposure to 5 mM MTZ of *Chipper* (Tg(*fli1a*:eGFP); Tg(*nphs2*:Eco.NfsB-mCherry); *mitfa*^*w2/w2*^*;  roy*^*a9/a9*^) while control larvae showed uniform covering with mCherry positive podocytes (scale bar represents 10 μm). The phenotype in transmission electron micrographs of 4 dpf *Cherry* larvae after 24 hours MTZ treatment verified these findings (panel b), as broad areas of the GBM (arrowheads) were denuded, while control larvae showed normal morphology with foot processes and filtration slits. Endothelial cells of denuded areas showed significant fewer fenestrations (arrows). Note the higher contrasted Bowman’s space in 5 mM MTZ group compared to control caused by proteinaceous fluid due to leakiness of the glomerular filter (scale bar represents 1 μm, yellow = GBM, representative images from n = 3 experiments).

After 24 h treatment of *Cherry* larvae (4 dpf) with either 5 mM MTZ or 0.1% DMSO (control), significantly fewer glomerular cells (0.02, SD = 0.002 versus 0.036, SD = 0.009 nuclei/μm^2^ of the glomerular area; p < 0.0001) were counted in cross sections of glomeruli of *Cherry* larvae which were imaged by confocal laser scanning microscopy (LSM); Supplementary Figure 2). To identify glomerular capillaries irrespective of the presence or absence of podocytes, we established a new transgenic zebrafish strain with additional fluorescently labeled endothelial cells. Therefore we crossbred the zebrafish strain Tg(*fli1a*:eGFP) which expresses eGFP in endothelial cells under control of the *fli1a* promotor with the *Cherry* strain and named the offspring *Chipper* (Tg(*fli1a*:eGFP); Tg(*nphs2*:Eco.nfsB-mCherry); *mitfa*^*w2/w2*^*; roy*^*a9/a9*^; Supplementary Figure 1). Supplementary Movie 1 shows an *in vivo* z-stack of a glomerulus of a *Chipper* larva (4 dpf). Cross sections of *Chipper* larvae after 16 hours treatment with MTZ showed capillaries, identified by the green fluorescence of the endothelial cells, with broad denuded areas, as indicated by the absence of red podocytes whereas the capillaries of control larvae were completely covered by mCherry expressing podocytes or their processes (Fig. 2a). To exclude that podocytes were still attached to the GBM but did not express mCherry due to the downregulation of podocin, we performed PAS and methylene blue staining of cross sections and transmission electron microscopy (TEM). The histological cross sections in Supplementary Figure 3a and b revealed areas along the capillaries which were not covered by podocytes in contrast to control larvae. Figure 2b and Supplementary Figure 3c show broad areas of denuded GBM (arrowheads in Fig. 2b), while glomeruli of control larvae showed an intact filtration barrier with interdigitating foot processes of podocytes covering the outer aspect of the capillaries (Fig. 2b).

Interestingly, the endothelial cells of denuded areas showed less fenestration compared to that in the control larvae (arrows in Fig. 2b). Additionally, MTZ-treated larvae showed a higher-contrasted Bowman’s space, presumably caused by the accumulation of proteins in the Bowman’s space due to a leaky filtration barrier (Fig. 2b).

### *In vivo* observation of the detachment of podocytes from the GBM

**Figure 3:**
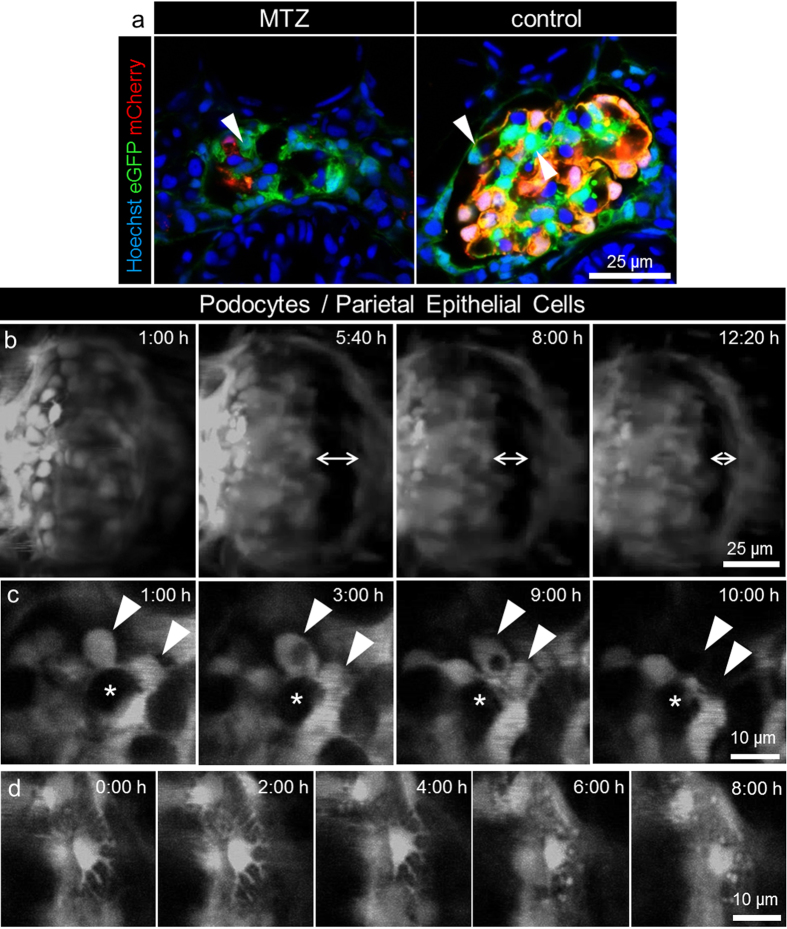
Long term *in vivo* two-photon imaging of podocyte injury. The pictures of panel a show that a subset of podocytes (arrowheads) in *Chet* larvae at 4 dpf only express eGFP and not NTR-mCherry and therefore are not vulnerable to MTZ treatment (representative image of n = 3 experiments, scale bar represents 25 μm). Panel b shows the morphological changes in the glomerulus during treatment with 5 mM MTZ as seen in the 3D reconstructions of long term 2-PM of *Chet* larvae beginning at 4 dpf. After approximately 5 hours, dilation of Bowman’s space occurred which decreased in the following time as shown by the double arrows at t = 5:40, 8:00, 12:20 hours (scale bar represents 25 μm). Panel c shows single frames of the detachment of two adjacent podocytes (arrowheads) between t = 9 and 10 hours (asterisks indicate a capillary loop which is covered by podocytes, scale bar represents 10 μm). The time series of single frames over 8 hours in panel d shows retraction of major processes of a single podocyte during treatment with 5 mM MTZ (scale bar represents 10 μm).

Since mCherry was not photo-stable enough for long-term observation by 2-PM (Supplementary Figure 4), we crossbred *Cherry* with the zebrafish strain *ET* (Tg(*wt1a*:eGFP); *mitfa*^*w2/w2*^*; roy*^*a9/a9*^)^1^. This new strain, named *Chet (**Ch**erry and **ET***; Tg(*nphs2*:Eco.NfsB-mCherry); Tg(*wt1a*:eGFP); *mitfa*^*w2/w2*^*; roy*^*a9/a9*^; Supplementary Figure 1), expresses eGFP in podocytes and parietal epithelial cells. Interestingly, not all podocytes expressed mCherry-NTR together with eGFP at 4 dpf (yellow, Fig. 3a). We found some podocytes which expressed eGFP but not mCherry (green, Fig. 3a, arrowhead) which were not affected by the MTZ treatment (MTZ-resistant).

To study the behavior of MTZ-resistant compared to MTZ-sensitive podocytes, we imaged up to 31 simultaneously embedded *Chet* larvae after the addition of MTZ over 24 hours. At the beginning (t = 0), we recorded a multitrack scan for eGFP and mCherry to verify the presence of mCherry-NTR-expressing podocytes. As a control we used *ET* larvae (control) which do not express mCherry-NTR. To exclude bleaching effects, we recorded z-scans only of the eGFP signal. The z-stack distance of the scans was 79 μm with a slice to slice distance of 1 μm. For spatiotemporal evaluation of the glomerular alterations, we reconstructed the z-volume of every point in time as 3D images and arranged them to 4D (3D over time) movies.

Supplementary Movie 2 shows a *Chet* glomerulus (4 dpf) treated with MTZ over a time period of 24 hours. As shown in Fig. 3b, we found that the fluorescence intensity and the number of MTZ-sensitive podocytes decreased during observation. Furthermore, a dilation of Bowman’s space was observed in 83.6% (SD = 25.7%, Supplementary Figure 5) of *Chet* larvae compared to 6.1% (SD = 3.9%) of control larvae, which started to resolve approximately at t = 12 hours (Fig. 3b, arrows).

After a few hours, we observed the detachment of podocytes. As shown in Supplementary Movie 2 most podocytes detached in clusters and in a synchronized fashion in each half of the pronephric glomerulus. In Fig. 3c, two neighboring podocytes detached from their capillary (Fig. 3c, asterisk) between t = 9 and 10 hours (Fig. 3c, arrowheads). In 77.1% (SD = 13.6%) of the larvae at least one of the remaining, non-detached podocytes retracted its major processes during the treatment with MTZ as shown in Fig. 3d and in Supplementary Movie 3.

Supplementary Movie 4 shows a 24 hours 4D movie of the glomerulus of a control larva (*ET)* during treatment with MTZ. As the podocytes did not express the NTR, they did not get affected by MTZ treatment, showed a stable eGFP expression and a normal phenotype.

### MTZ treatment induces pseudocyst formation in the subpodocyte space

**Figure 4:**
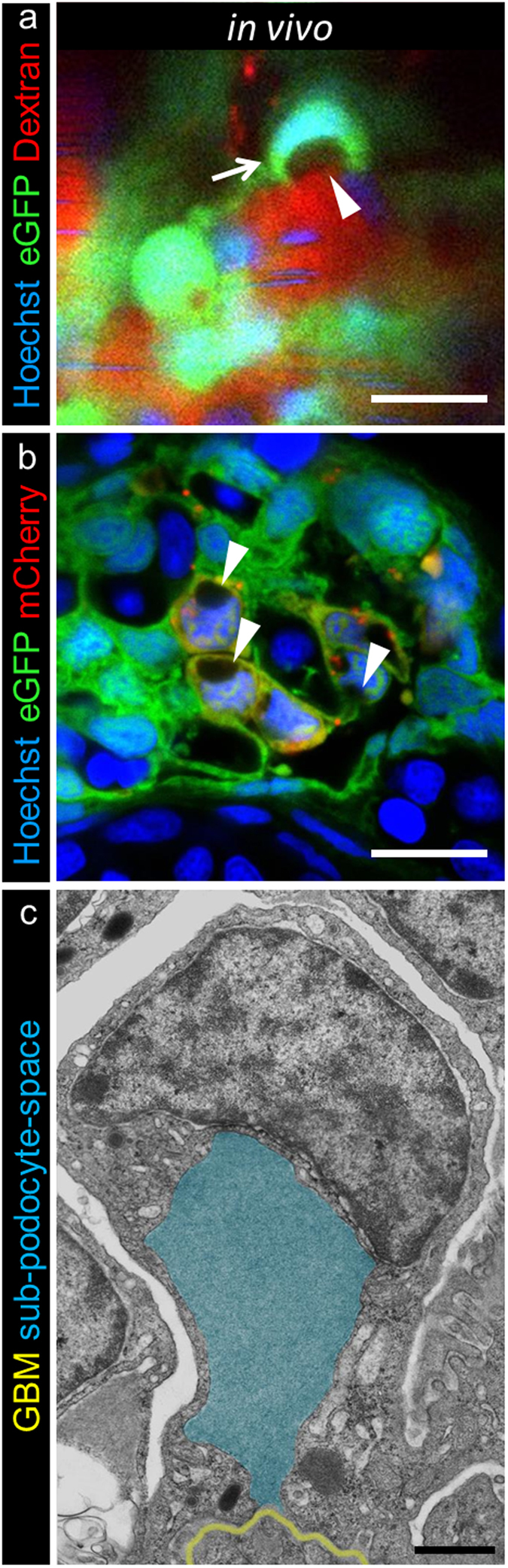
Subpodocyte space pseudocysts form during treatment with MTZ. 2-PM of 0.5 mg/ml Hoechst 33342 and 25 mg/ml TRITC-dextran injected *Chet* larvae at 5 dpf revealed pseudocysts (**a**, arrowhead, scale bar represents 10 μm) within the cell bodies and nuclei that were pushed aside (**a**). The parachute-like pseudocysts have a basal opening to the GBM and are encapsulated by podocyte cell bodies (**a**, arrow). The striped Hoechst signal in capillaries is due to blood flow of nucleated erythrocytes in living larvae (representative images of n = 4 individual experiments, scale bar represents 5 μm). The appearance of pseudocysts was consistent with findings in confocal laser scanning microscopy of cross sections after 3 hours 5 mM MTZ treatment of 4 dpf *Chet* larvae as seen in picture (**b**), while control treated larvae did not show any pseudocysts (Supplementary Figure 6b,c, representative images of n = 3 individual experiments). Transmission electron microscopy further revealed the ultrastructural composition of these pseudocysts as exemplarily shown in picture c. Injured podocytes showed a basal opening to the GBM (yellow) and large pseudocysts (blue) which were encapsulated by the podocyte cell bodies (scale bar represents 1 μm, representative image from n = 3 experiments).

During MTZ treatment we observed growing “holes” in or near the podocyte cell bodies of *Chet* larvae. To characterize these structures, we injected a mixture of 0.5 mg/ml Hoechst 33342 (to label the nuclei) and 25 mg/ml TRITC labeled 2000 kDa Dextran (to label the blood plasma). After the treatment of the larvae with MTZ for 3 hours, pictures were acquired by 2-PM. Figure 4 a shows that the podocyte nuclei (in blue) are pushed to the apical part of the cell. Surprisingly, many, but not all, “holes” were filled with TRITC-dextran (Supplementary Figure 6a), indicating a dilated subpodocyte space with connection to the denuded GBM. To verify this hypothesis, we further studied cross sections of these larvae by LSM which confirmed the position of the dilations and displaced nuclei (Fig. 4b, Supplementary Figure 6b). The same structures were found in methylene blue stained sections as seen in Supplementary Figure 6c (askerisks). By TEM, we found parachute-like shaped podocytes with a basal opening to the GBM, dislocated nuclei and basal pseudocysts filled with rather electron dense material (Fig. 4c).

**Figure 5:**
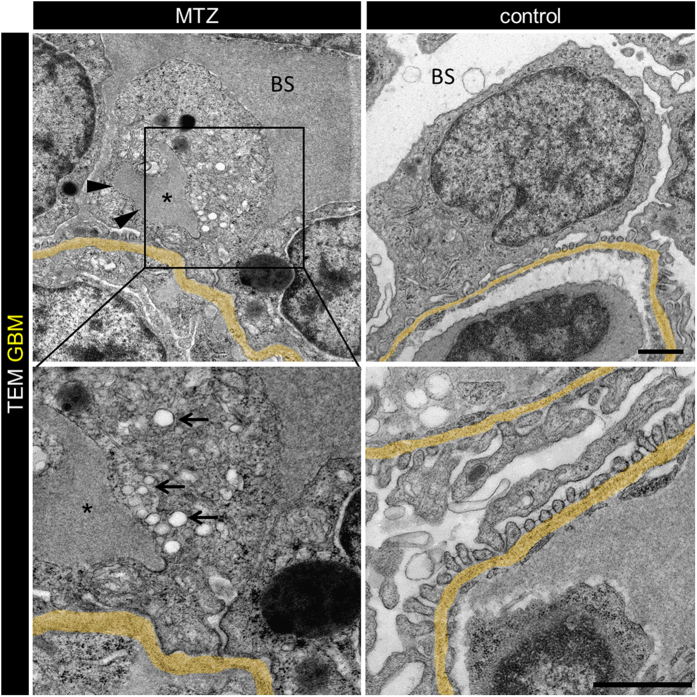
Injured podocytes show increased endocytosis from subpodocyte space dilatations. Transmission electron micrographs of 3 hours 5 mM MTZ treated *Cherry* larvae showed appearance of pseudocysts (arrowheads) and numerous endosomes (arrows) close to the pseudocysts (lumen marked with asterisk). Additionally those larvae showed broad effacement of foot processes. In contrast, control treated larvae (0.1% DMSO) showed normal glomerular morphology with regular foot processes and slit diaphragms in between. Note the electron dense Bowman’s space (BS) in MTZ treated larvae (scale bars represent 1 μm, representative images from n = 3 experiments).

Furthermore, we found numerous endosomes that were located in close vicinity to these pseudocysts, while the control larvae did not show increased numbers of endosomes in the region of the subpodocyte space (Fig. 5). Additionally, we found that, already after 3 hours MTZ treatment, Bowman’s space appeared to be significantly electron-denser compared to control larvae presumably due to leakiness of the glomerular filtration barrier (Fig. 5, marked by BS).

### Podocytes do not migrate along the naked GBM

To exclude MTZ targeted ablation of potential progenitor cells through upregulation of the *nphs2* promotor followed by the expression of the NTR, we performed a second 24 hours long term imaging experiment with n = 31 *Chet* larvae (4 dpf) which were pretreated with MTZ for 3 hours (n = 17) or control (n = 14) followed by three times washing in E3 medium and transfer to 2-PM. These pretreated larvae showed the same phenotype like the larvae which were continuously treated with MTZ simultaneous to 2-PM.

**Figure 6:**
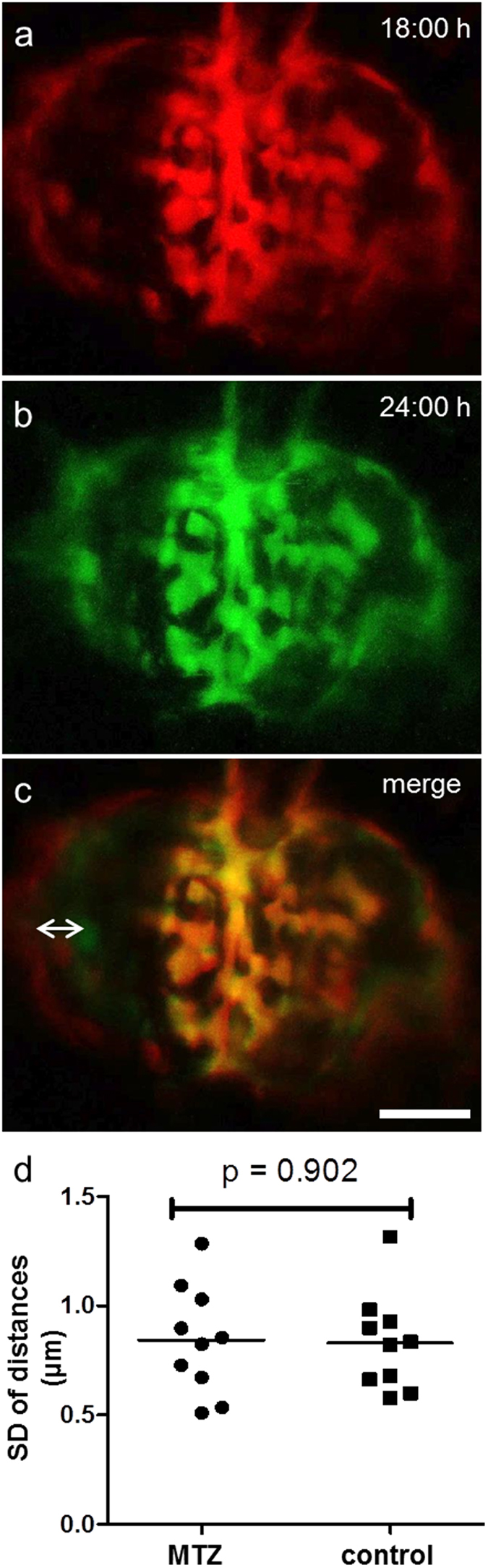
Migration-analysis reveals static behavior of remaining podocytes. For evaluation of possible migration we captured 3D images of different points in time as seen for t = 18 hours (**a**) and t = 24 hours (**b**). The merged pictures in c show that the parietal epithelial cells do not match (double arrow) as Bowman’s space edema resolves over time. In contrast, podocytes on the glomerular tuft show no change of their position between the single points in time (scale bar represents 25 μm). The diagram in d shows the mean standard deviations of the podocytes distances measured over 15 hours for up to 18 different time points. The mean SD was 0.84 μm for MTZ and 0.83 μm for the DMSO control group (n = 10 per group). There was no significant difference between groups (p = 0.902).

To elucidate the question of potential podocyte migration on the denuded GBM, we performed two different experiments. First, we compared the pattern of remaining podocytes by overlaying 3D pictures as seen in Fig. 6a–c for t = 18 hours and t = 24 hours. While positions of parietal epithelial cells differed due to the decreasing volume of Bowman’s space edema as indicated by the double arrow in Fig. 6c, the patterns of the glomerular tuft matched as seen in the merged image in Fig. 6c. Second, to confirm the static behavior of podocytes as seen in our previous findings, we measured the distances between podocytes over 15 hours in MTZ-treated (3 hours) and control larvae (each n = 10). The diagram in Supplementary Figure 7 shows the distances between two podocytes of 8 out of 20 larvae that were measured. We found that the mean standard deviations were 0.84 μm (SD = 0.23) for MTZ-treated and 0.83 μm (SD = 0.21) for control larvae (Fig. 6d). Since there was no significant difference between the groups (p = 0.902, Student’s *t*-test) and the standard deviation of the change in distances was below the resolution limit of 1 μm we concluded that there was no lateral movement of podocytes along the naked GBM within the first 24 hours of acute podocyte injury in the pronephric glomerulus.

## Discussion

The zebrafish model offers many advantages over other animal models of podocyte injury. While rodent based *in vivo* imaging procedures are quite expensive, labor intensive and time-consuming, zebrafish are easy to breed and handle. Additionally, this model can be easily applied to high-throughput experiments, which makes it an interesting tool for drug screenings. Earlier work has demonstrated the applicability of the NTR/MTZ model to the analysis of new proteins (e.g. through transgenic overexpression, knockout, or morpholino-mediated knockdown) associated with podocyte injury^8^,^9^. These models can easily be applied to our semi-automated imaging procedure delivering an additional *in vivo* morphology aspect and exciting opportunities for high-throughput analysis.

While the original NTR/MTZ strain was limited to microscopic endpoint analysis^6^,^7^, we are now able to continuously track morphological alterations in the *Cherry, Chet* and *Chipper* strains by 2-PM during the progression of podocyte injury. Analog to earlier findings for the Tg(*nphs2*:Eco.nfsB-mCherry) strain, treatment with MTZ led to formation of pericardial and periocular edema, apoptosis and podocyte injury in the new transparent *Cherry* strain. Podocyte slit diaphragm proteins were significantly downregulated as demonstrated by (q)RT-PCR and immunostainings. While podocin is exclusively expressed in podocytes, there is an additional expression of nephrin in the brain, pancreas, and in developing myoblasts^10^,^11^. Therefore the decrease in *nphs1* expression after MTZ treatment as shown by qRT-PCR is smaller compared to the massive decrease in the expression of *nphs2*.

Enlargement of the subpodocyte space, often described as pseudocysts or ‘vacuolization’ of podocytes, is a frequently observed feature of podocytes that is thought to precede detachment^12^,^13^,^14^,^15^. Broadening and fusion of the major processes restrict the efflux of filtrate from the subpodocyte space resulting in the formation of pseudocysts^15^. Pseudocyst formation during MTZ exposition in the zebrafish recapitulates a characteristic finding of injured podocytes. Therefore, it appears very unlikely that pseudocyst formation is a peculiar phenomenon of the MTZ model unrelated to glomerular disease. Our experiments showed that, while some pseudocysts accumulated dextran, others had similar fluorescence intensities like Bowman’s space. These results indicate that there are connections between the subpodocyte space and Bowman’s space which vary in their permeability.

The enriched amount of endosomes we found adjacent to the pseudocysts strengthens the theory of transcytosis from the subpodocyte space by podocytes as previously investigated for labeled albumin in a 2-PM study in rats by Schießl and colleagues^16^.

Besides these common features of acute podocyte injury, our 2-PM data indicate that pronephric podocytes in zebrafish larvae detach in clusters from the GBM (Supplementary Movie 2). This phenotype has been described before in the pathogenesis of acute podocyte injury in rodents and suggests a conserved sequence of podocyte detachment over the different species^14^.

As the electron micrographs showed a significantly electron denser Bowman’s space after 3 and 24 hours MTZ treatment we conclude that the glomerular filter got leaky as early as 3 hours after the onset of MTZ treatment. This finding confirms that already early alterations of MTZ-induced podocyte injury lead to proteinuria as shown before using *in vivo* 2-PM in a zebrafish strain which endogenously expresses a fluorescence labeled filtration marker^17^.

Additionally, we found significantly fewer fenestrations of endothelial cells adjacent to podocyte-denuded GBM. A similar phenotype, with loss of fenestrations and swollen cellular bodies, has been described in glomerular endothelial cells of podocyte specific VEGF knockout or knockdown mice^18^,^19^. This underlines the importance of intact podocytes and their VEGF-A secretion for the homeostasis of glomerular endothelial cells.

Based on the ability of cultured podocytes to migrate *in vitro*, it was suggested that podocytes *in vivo* migrate along the GBM in a lateral fashion, especially in podocytopathies as demonstrated by Peti-Peterdi and colleagues by 2-PM in PAN-treated rats^20^,^21^. In a recent *in vivo* and *ex vivo* 2-PM study, Brähler and colleagues described augmented membrane dynamics, retraction of major processes and detachment of single podocytes, but no lateral migration in a murine constitutively active Rac1 expression model as well as in mice which were injected with a nephrotoxic serum^2^. As described in the aforementioned work, we have found loss of major processes and detachment of podocytes from the capillaries during the progression of acute podocyte injury, resembling the human acute podocyte injury. However, we like to mention that the behavior of podocytes might be different in mammals or even in a different zebrafish injury model.

As our resolution limit during 2-PM is about 1 μm, we are not able to track changes of the foot processes, which may also result in podocyte spreading. However, our work gives strong evidence that even in the absence of continuous covering of the GBM there is no lateral migration of podocytes along the denuded GBM within the early time course of 24 hours.

Taken together, our results indicate that even after the induction of acute podocyte injury and partial denudation of the GBM, pronephric podocytes do not begin to migrate along the GBM making regenerative processes through the immigration of progenitor cells inside or outside the glomerulus within the first 24 h very unlikely.

## Methods

### Zebrafish Stock

Zebrafish stocks and larvae were maintained as described previously^22^. The *Cherry* (Tg(*nphs2*:Eco.NfsB-mCherry); *mitfa*^*w2/w2*^*; roy*^*a9/a9*^) strain expresses the prokaryotic enzyme nitroreductase under control of the podocyte specific *nphs2* promotor in the transparent *Casper (mitfa*^*w2/w2*^*; roy*^*a9/a9*^)^23^ background. For fluorescence labeling of the endothelium we crossbred *Cherry* (Tg(*nphs2*:Eco.NfsB-mCherry); *mitfa*^*w2/w2*^*; roy*^*a9/a9*^) with *Flipper* (Tg(*fli1a*:eGFP); *mitfa*^*w2/w2*^*; roy*^*a9/a9*^), which expresses eGFP in endothelial cells, selected for double fluorescence and named the offspring *Chipper* (Tg(*fli1a*:eGFP); Tg(*nphs2*:Eco.nfsB-mCherry); *mitfa*^*w2/w2*^*; roy*^*a9/a9*^). The strains *ET* (Tg(*wt1a*:eGFP); *mitfa*^*w2/w2*^*; roy*^*a9/a9*^)^1^ and *Cherry* were crossbred, double transgenic offspring selected, reared to adulthood and named *Chet* (Supplementary Figure 1).

All experiments were performed in accordance with German animal protection law overseen by the “Landesamt für Landwirtschaft, Lebensmittelsicherheit und Fischerei, Rostock” of the federal state of Mecklenburg - Western Pomerania. All 2-PM experiments were performed at 22 °C. MTZ (Sigma-Aldrich, St. Louis, MO, USA) was freshly diluted in 0.1% DMSO-E3 medium. MTZ treatment with a concentration of 10 mM was used for verification of apoptosis by TUNEL assay and gene expression analysis, all other experiment were conducted with 5 mM MTZ.

### *In vivo* imaging

For *in vivo* imaging zebrafish larvae were embedded in 0.8% low-melting agarose (Biozym LE agarose, Germany) in a dorsal side up position on the cover of a 96-well plate. After hardening the larvae were covered with E3 medium supplemented with 5 mM MTZ and 0.06 mg/ml tricaine (Sigma-Aldrich). MTZ was dissolved in 0.1% DMSO in E3 medium. For intravenous injection of 0.5 mg/ml Hoechst 33342 (Sigma-Aldrich) and 25 mg/ml TRITC labeled 2000 kDa dextran (Thermo-Fisher Scientific, Waltham, MA, USA), larvae were anaesthetized in 0.08 mg/ml tricaine in E3 medium and injected with about 3 nl using a microinjector (transjector 5246; Eppendorf AG, Hamburg, Germany) with specialized glass capillaries (Femtotips; Eppendorf AG). 2-PM was performed with a LSM710MP (Carl Zeiss Microimaging, Jena, Germany) and a Chameleon Ti-Sapphire Laser (Chameleon, Coherent, Santa Clara, CA, USA). Excitation wavelengths were 860 nm for eGFP, TRITC, Hoechst 33342 and 760 nm for mCherry. For multi position long term imaging up to 31 larvae were imaged subsequently on every point in time. Z-stacks of glomeruli were recorded over a distance of 79 μm with a voxel volume of 0.16 × 0.16 × 1 μm and reconstructed as 3D images which then were arranged as 4D movies and drift-corrected with ImageJ (NIH, Bethesda, USA). For co-localization analysis 3D pictures of different points in time were exported, position-corrected and merged with GNU Image Manipulation Program 2.8 (The GIMP Development Team, USA). Distances between cell bodies of podocytes were measured for up to 18 points in time with the ZEN 2010 software (Carl Zeiss Microimaging, Jena, Germany).

### Histology

For immunofluorescence staining the larvae were fixed in 2% paraformaldehyde in 1x PBS buffer for 3 hours at room temperature followed by infiltration with 30% sucrose in PBS at 4 °C overnight. Larvae were placed in Tissue Tek embedding medium (Sakura, Staufen, Germany), snap frozen in liquid nitrogen and cut on a Microm HM 560 microtome (Thermo Fisher Scientific). For immunostaining, the sections were incubated with primary antibodies 1:2000 rabbit anti-nephrin (Innovagen, Lund, Sweden) or 1:700 rabbit anti-podocin (Proteintech, IL, USA) at 4 °C overnight. After three washes in 1x PBS and incubation with the Alexa 488 conjugated goat anti-rabbit F(ab) antibody fragment (1:250; Dianova, Hamburg, Germany) the slides were incubated with 0.013 mg/ml Hoechst 33342 (Sigma-Aldrich). After several washes in 1x PBS the slides were mounted in Mowiol for microscopy (Carl Roth, Karlsruhe, Germany). For TUNEL assay we used the *in situ* Cell Death Detection Kit (Roche, Basel, Switzerland) according to the manufacturer’s description. The stained sections were imaged using a TCS SP5 confocal laser scanning microscope system (Leica Microsystems, Wetzlar, Germany). The number of cell nuclei and the glomerular area were measured using the cell-counter plugin of ImageJ (NIH, Bethesda, USA).

### Transmission electron microscopy

Larvae at 4 dpf were fixed in 4% glutaraldehyde, 1% paraformaldehyde and 1% sucrose in 0.1 M HEPES for 3 hours on ice. After postfixation in 2% osmium tetroxide and dehydration in ethanol the larvae were embedded in EPON 812 (Serva, Heidelberg, Germany). Semithin (500 nm) and ultrathin sections (70 nm) were cut on an Ultracut UCT ultramicrotome (Leica Microsystems, Heidelberg, Germany). Semithin sections were stained with methylene blue. UItrathin sections were placed on copper grids, contrasted with 5% uranyl acetate for 5 min and with Sato’s lead stain for 5 min. Images were acquired with a LIBRA 120 transmission electron microscope (Carl Zeiss GmbH, Oberkochen, Germany).

### RNA isolation and (q)RT-PCR

RNA of 15–20 decapitated larvae was isolated using TRI-Reagent (Sigma-Aldrich) according to the manufacturer’s description followed by cDNA synthesis with QuantiTect reverse transcription kit (Qiagen, Hilden, Germany). RT-PCR was carried out with peqGOLD Taq-DNA polymerase (VWR, Erlangen, Germany). Quantitative RT-PCR was performed with SYBR green master mix (BioRad, Hercules, USA) on an iCycler Thermal Cycler (BioRad, Hercules, USA). Primer sequences and cycler programs can be found in the Supplementary information. Expression calculation was performed using the ΔΔCt method and were normalized with control (0.1% DMSO treated) group.

### Statistical analysis

Gaussian distribution was checked by Kolmogorov-Smirnov testing, followed by significance testing with Student’s t -test using SPSS V.22 (IBM, Armonk, NY, USA). P-values below 0.05 were declared as statistical significant. For statistical testing of non-parametric data of the cell nuclei density measurements, the Mann-Whitney *U*-test was applied.

## Additional Information

**How to cite this article**: Siegerist, F. *et al*. Acute podocyte injury is not a stimulus for podocytes to migrate along the glomerular basement membrane in zebrafish larvae. *Sci. Rep.* **7**, 43655; doi: 10.1038/srep43655 (2017).

**Publisher's note:** Springer Nature remains neutral with regard to jurisdictional claims in published maps and institutional affiliations.

## Supplementary Material

Supplementary Information

Supplementary Movie 1

Supplementary Movie 2

Supplementary Movie 3

Supplementary Movie 4
