# Supplementary material for: Acute podocyte injury is not a stimulus for podocytes to migrate along the glomerular basement membrane in zebrafish larvae
Source: Sci Rep. 2017 Mar 2;7:43655. doi: 10.1038/srep43655 (PMC5333633; doi:10.1038/srep43655)
Supplement: Supplementary Information [file srep43655-s1.pdf]

Acute podocyte injury is not a stimulus for podocytes to migrate along the glomerular basement membrane in zebrafish larvae

Florian Siegerist[1], Antje Blumenthal[1], Weibin Zhou[2], Karlhans Endlich[1], Nicole Endlich[1*]

[1]*Department of Anatomy and Cell Biology, University Medicine Greifswald, Greifswald, Germany*

[2]*Department of Pediatrics and Communicable Diseases, University of Michigan, Ann Arbor, Michigan, USA*

Supplementary Material and Methods

qRT-PCR

Primer	Sequence
nphs1 F	CAATGTCCCTAACCCGCACT
nphs1 R	ACGCCTCACATTGCAGAGAA
nphs2 F	GGCCCTGGGCTGATGTTTTA
nphs2 R	GAGCAATGCGTTTCCTGTCC
e*ef1a1l1* F	AAGGAGGGTAATGCTAGCGG
e*ef1a1l1* R	GGGCGAAGGTCACAACCATA
zgc:158463 F	TTACCCCAGGCTCGGAAAAC
zgc:158463 R	CGGGAAGGTCTTTGAACCCA

Cycler program

**Cycle #**	**Step**	**Temperature in °C**	**Duration in s**
**1**	1	95	300
**2**	1	95	15
	2	60	25
	3	72	20
**3**	1	95	60
**4**	1	55	1

Steps 2-1, -2, -3 were repeated 50 times and followed by a melting curve up to 95°C. The primers were validated in a conventional end point RT-PCR and sequencing of the PCR products.

Technovit 7400 Embedding and PAS staining

Zebrafish larvae were fixed 2% PFA for 3 hours at room temperature with gentle agitation. After an ascending ethanol series the Technovit 7400 (Haereus Kulzer GmbH, Wehrheim, Germany) embedding was performed according to the manufacturer's protocol. After polymerization, 3 µm sections were cut on a Leica rotational microtome (Jung RM2055, Leica Biosystems, Wetzlar, Germany). For PAS staining, the slides were incubated for 15 min in 2% sodium metabisulfite, washed in Aqua dest followed by 30 min incubation in 1% periodic acid at 56°C. After rinsing in Aqua dest, the slides were incubated for 25 min in Schiff reagent, rinsed in tap water and Aqua dest. For nuclear counterstaining, the slides were incubated for 15 min in Gill's haematoxylin, rinsed in tap water followed by an ascending ethanol series, clearing in xylene and mounting in Eukitt.

Supplementary Figures

Supplementary Figure 1

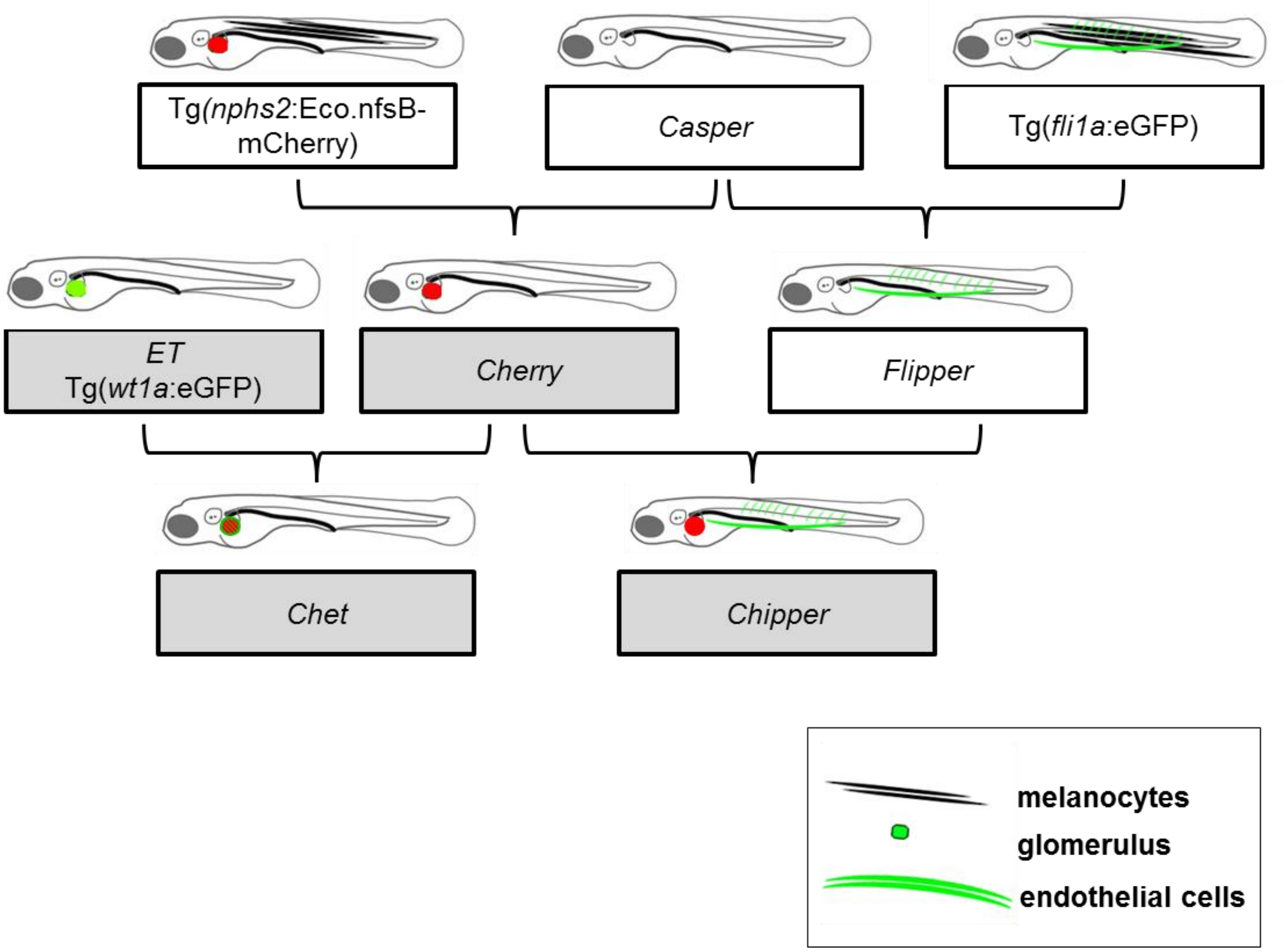

Supplementary Figure 1 shows the breeding scheme of this study. The strains that are labeled in grey were used for experiments.

Supplementary Figure 2

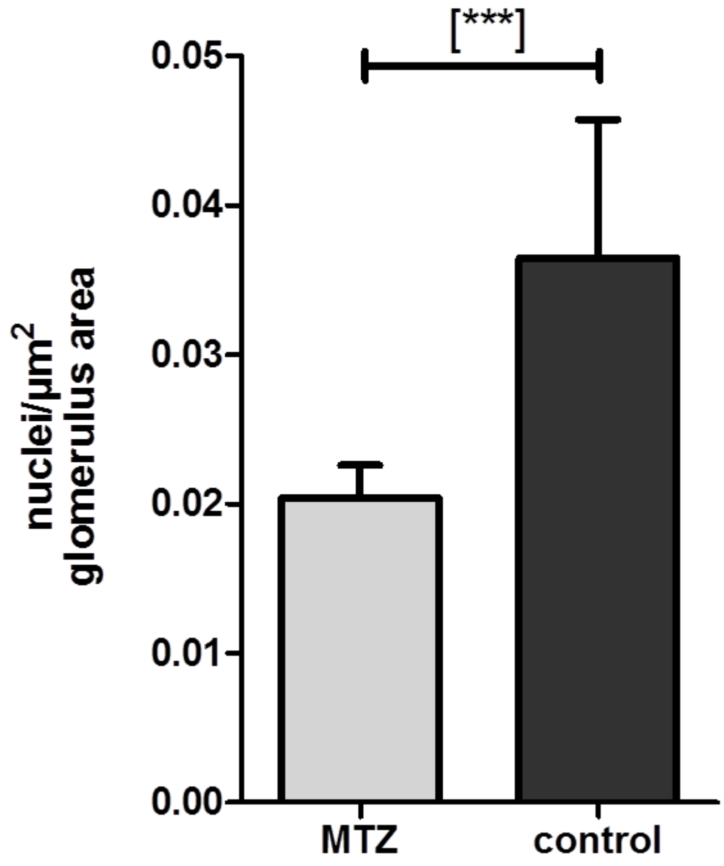

Supplementary Figure 2 shows the number of cell nuclei per µm^2 glomerulus area. Compared to control-treated (0.1% DMSO, n=10 larvae, 0.036 nuclei/µm^2, SD=0.01), 5mM MTZ treated *Cherry* larvae showed significantly fewer (0.02 nuclei/µm^2, SD=0.002, p>0.0001) cell nuclei per µm^2 glomerular area.

Supplementary Figure 3

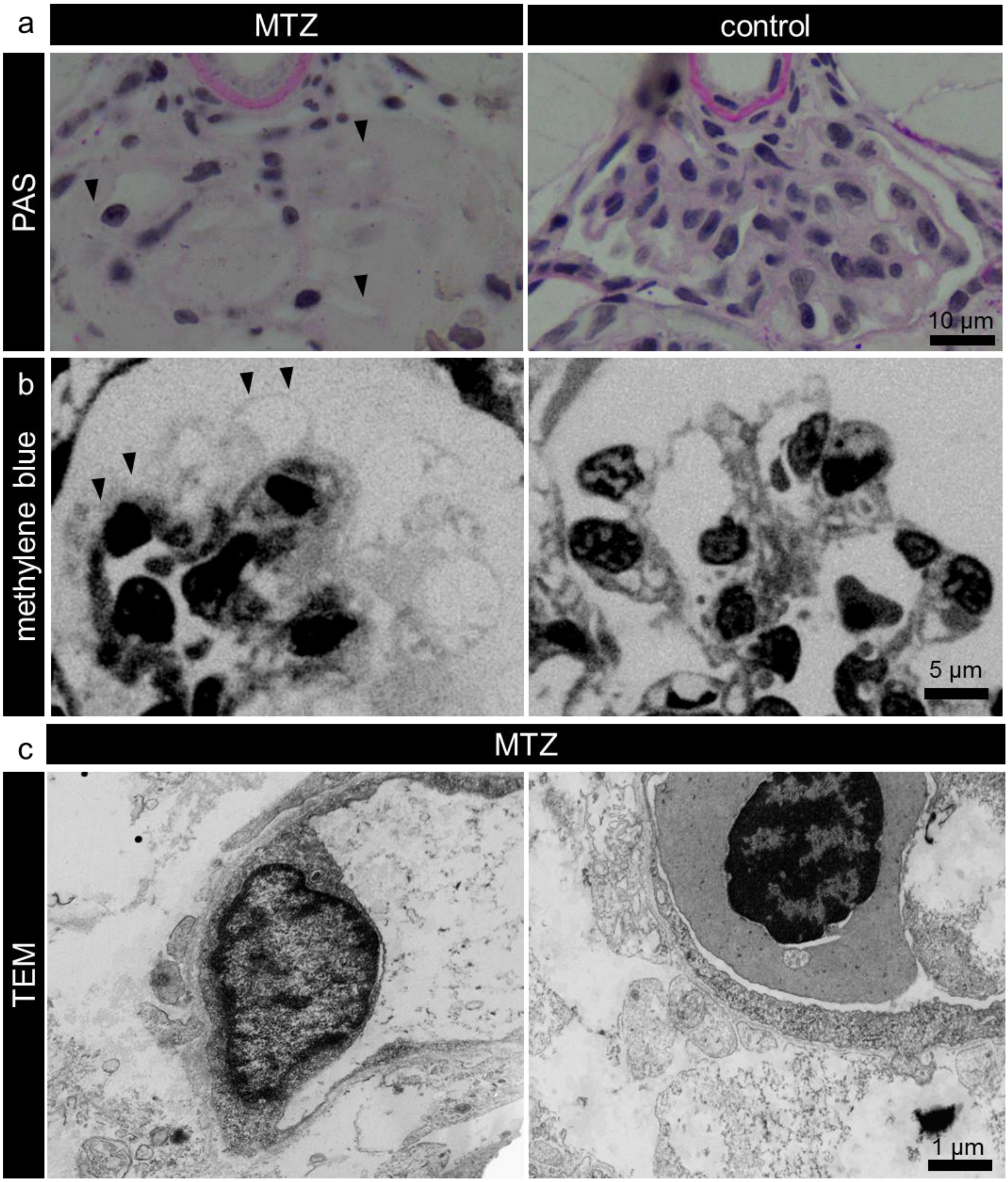

Supplementary Figure 3 shows the denudation of the GBM. The PAS stained sections in panel a shows that broad areas of the glomerular capillaries were denuded and not covered by podocytes (a, arrowheads). Methylene blue stained semithin sections (panel b) showed absence of podocytes from the glomerular capillaries (b, arrowheads). Panel c shows additional transmission electron micrographs to Figure 2 b.

Supplementary Figure 4

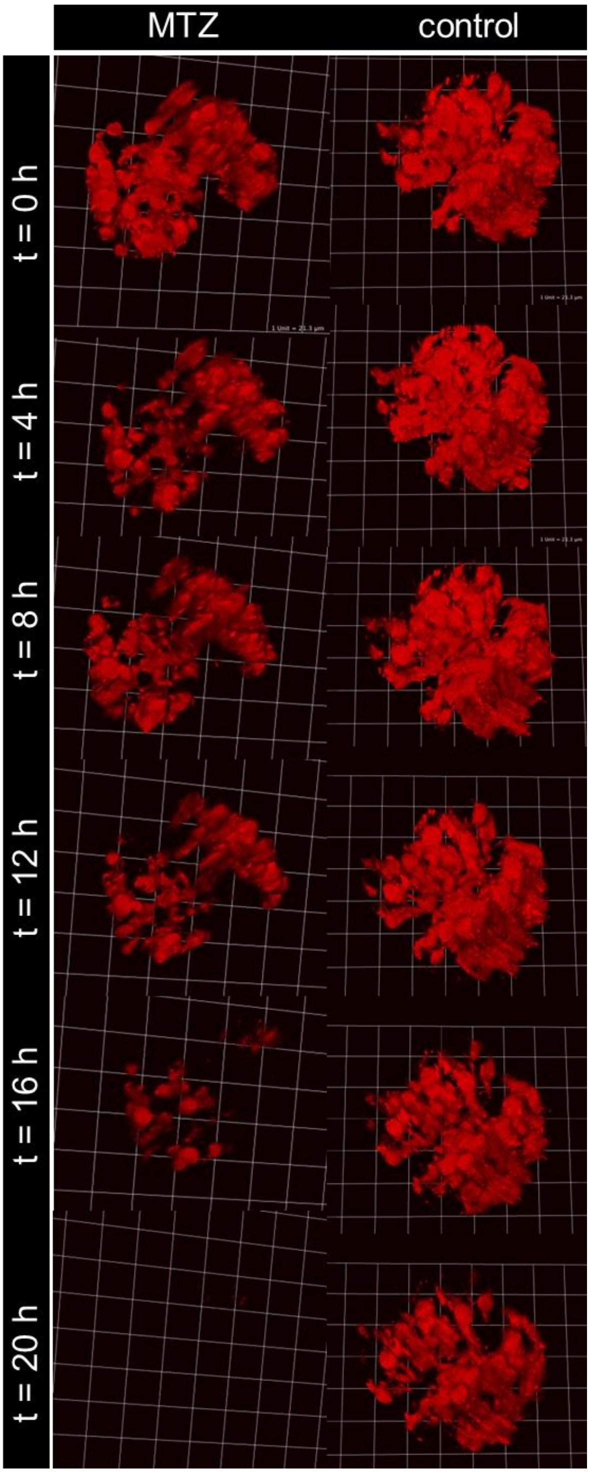

Supplementary Figure 4

20 hours treatment with MTZ of 4 dpf Cherry larvae during 2-PM leads to massive loss of mCherry positive podocytes as indicated by the decrease of fluorescence. Besides that, the fluorescence of control (0.1% DMSO) also decreases due to bleaching effects because of the relatively short two-photon excitation wavelength of 760 nm.

Supplementary Figure 5

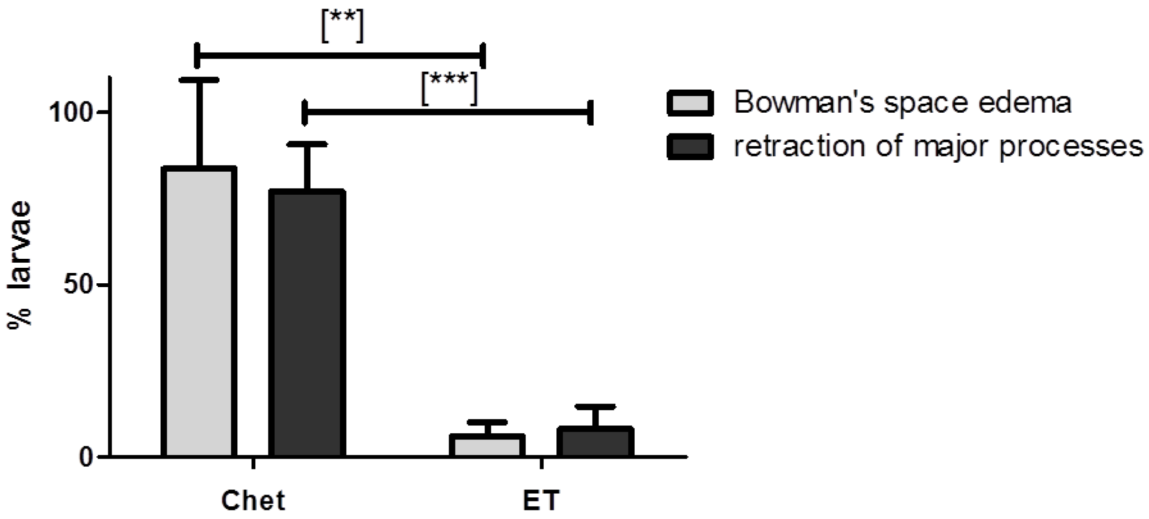

Supplementary Figure 5

During 24 hours 2-PM observation of MTZ-induced podocyte injury, 83.6% (SD=25.7%) of Chet (n=61 in three individual experiments) and 6.1% (SD=3.4%) of ET larvae (n=49 in three individual experiments) developed Bowman's space edema. Retractions of major processes could be captured in 77.1% (SD=Chet) versus 8.2% (SD=6.5% ET) of the larvae. [**]:p<0.01; [***]:p<0.001, error bars indicate SD.

Supplementary Figure 6

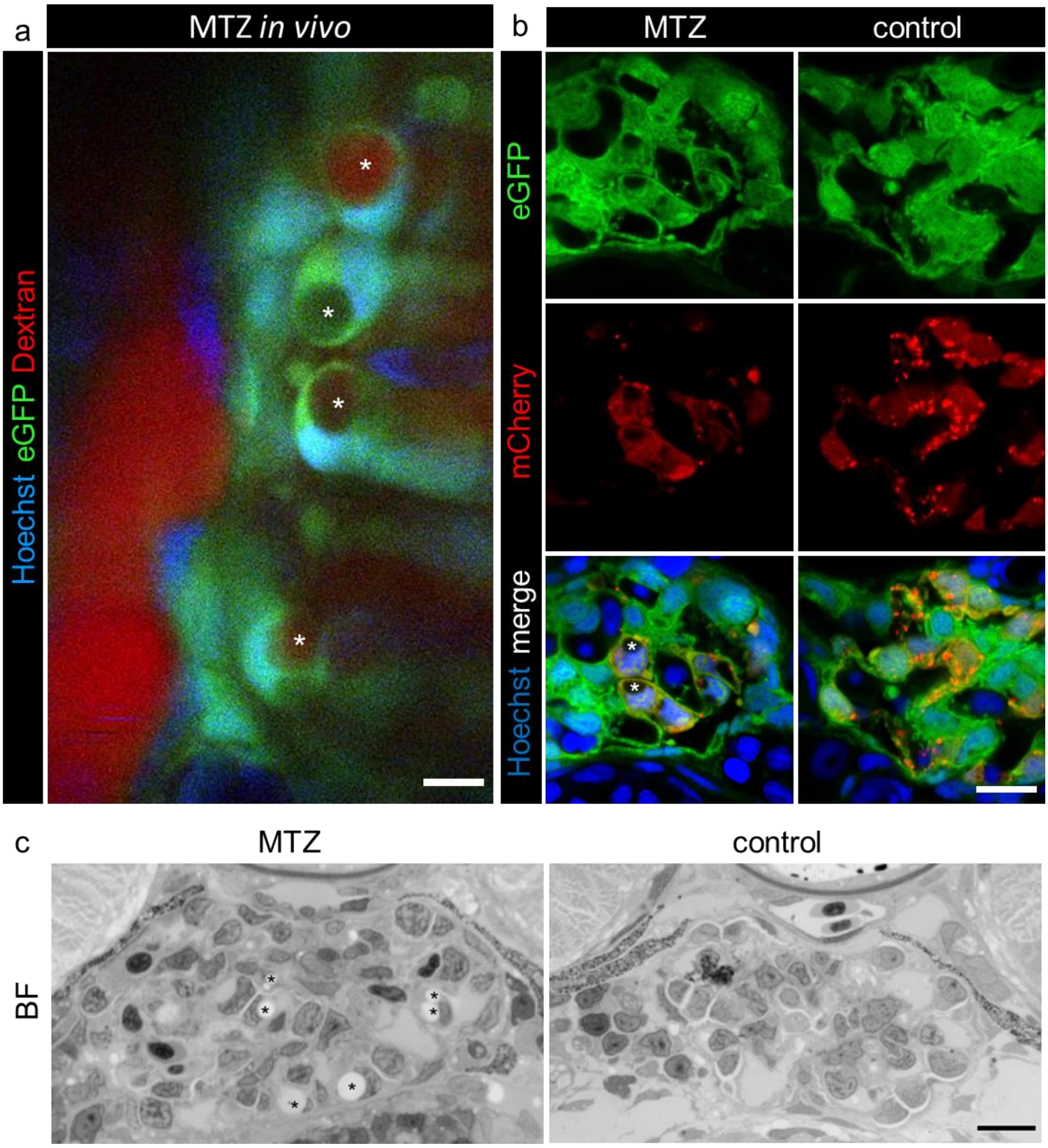

Supplementary Figure 6

Picture a shows a 2-PM of a *Chet* larva which was pre-treated with MTZ for 3 hours. A few pseudocysts (asterisks) can be distinguished with different dextran fluorescence intensities (red, scale bar represents 10 µm). Panel b shows the control confocal laser scanning micrographs to Figure 4 b. Compared to MTZ treated larvae, controls did not show pseudocysts and normal glomerular morphology (scale bar represents 5 µm). The pictures of the methylene blue stained semithin sections in panel c confirm the appearance of these pseudocysts, as multiple encapsulated holes (asterisks) could be distinguished in MTZ treated larvae, whereas control glomeruli looked normally (scale bar represents 10 µm).

Supplementary Figure 7

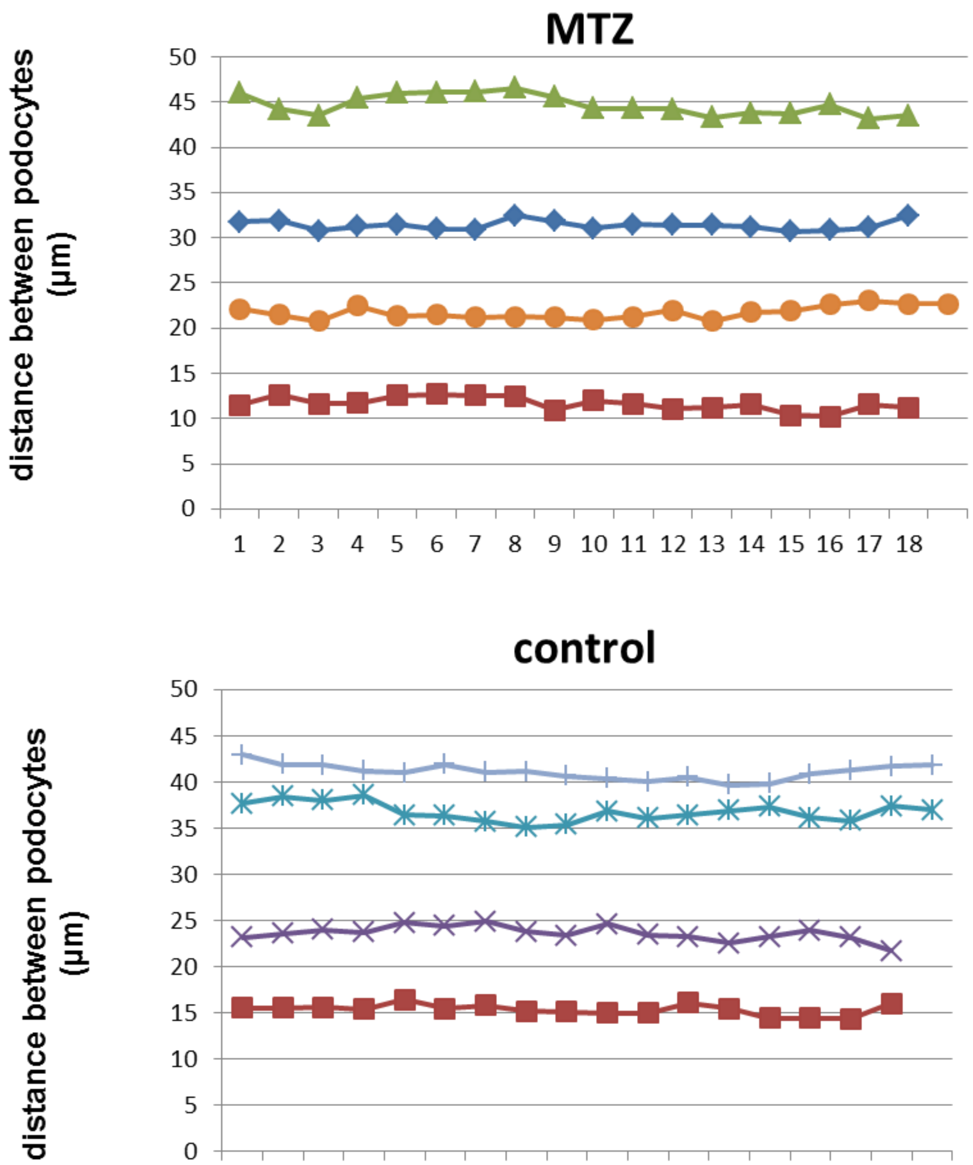

Supplementary Figure 7

The line diagram shows the distances between podocytes measured by 2-PM on 18 points in time over 15 hours in 3 hours MTZ pretreated or control larvae. There is no directed change of distance as well as no significant difference in the slope between the groups.

Supplementary Movies

Supplementary Movie 1

Supplementary Movie 1 shows a 2-PM z-stack of a *Chipper* larva at 4 dpf. The mCherry expressing podocytes (red) can be distinguished in their regular position on the GBM opposite to the eGFP expressing glomerular endothelial cells.

Supplementary Movie 2

Supplementary Movie 2 shows the glomerular alterations during 24h MTZ (5 mM) treatment in a *Chet* larva as a 4D time lapse movie reconstructed from 2-PM z-stacks of the complete glomerulus. The movie shows a massive decrease of the eGFP expression and detachment of podocytes from the glomerular capillaries. Additionally, the appearance of pseudocysts can be seen during MTZ the treatment prior to detachment.

Supplementary Movie 3

Supplementary Movie 3 shows the retractions of the major processes of a single podocyte during the treatment with of a *Chet* larva with 5 mM MTZ as 3D movies of different points in time reconstructed from multiple 2-PM z-stacks.

Supplementary Movie 4

Supplementary Movie 4 shows a NTR negative *ET* control larva, treated with 5 mM MTZ. Over the time course, the podocytes remain static and compared with Supplementary Movie 2 (*Chet*), the fluorescence intensity remains stable and no podocytes detach from the GBM.
